# Elucidating the Chemistry behind the Reduction of Graphene Oxide Using a Green Approach with Polydopamine

**DOI:** 10.3390/nano9060902

**Published:** 2019-06-21

**Authors:** Cláudia Silva, Frank Simon, Peter Friedel, Petra Pötschke, Cordelia Zimmerer

**Affiliations:** Leibniz Institute of Polymer Research Dresden (IPF), 01069 Dresden, Germany; frsimon@ipfdd.de (F.S.); friedel@ipfdd.de (P.F.); poe@ipfdd.de (P.P.)

**Keywords:** graphene oxide, reduced graphene oxide, X-ray photoelectron spectroscopy, Raman spectroscopy, electrical conductivity, functionalization

## Abstract

A new approach using X-ray photoelectron spectroscopy (XPS) was employed to give insight into the reduction of graphene oxide (GO) using a green approach with polydopamine (PDA). In this approach, the number of carbon atoms bonded to OH and to nitrogen in PDA is considered and compared to the total intensity of the signal resulting from OH groups in polydopamine-reduced graphene oxide (PDA-GO) to show the reduction. For this purpose, GO and PDA-GO with different times of reduction were prepared and characterized by Raman Spectroscopy and XPS. The PDA layer was removed to prepare reduced graphene oxide (RGO) and the effect of all chemical treatments on the thermal and electrical properties of the materials was studied. The results show that the complete reduction of the OH groups in GO occurred after 180 min of reaction. It was also concluded that Raman spectroscopy is not well suited to determine if the reduction and restoration of the sp^2^ structure occurred. Moreover, a significant change in the thermal stability was not observed with the chemical treatments. Finally, the electrical powder conductivity decreased after reduction with PDA, increasing again after its removal.

## 1. Introduction

Graphene, a 2D monolayer of sp^2^-hybridized carbon atoms arranged in a hexagonal lattice with a carbon–carbon bond length of 0.142 nm, has been extensively studied since it was first isolated in 2004 by Novoselov et al. [1,2]. It has a great potential for several applications due to its Young’s modulus of 1 TPa, intrinsic strength of 130 GPa [3], room temperature (RT) electron mobility of 250,000 cm^2^ V^−1^ s^−1^ [4], and optical transmittance of 97.7% [5]. The promising application areas for graphene are photonics, optoelectronics, energy generation and storage, sensors for gas detection, reinforcement of composite materials, and biomedical areas, particularly in biosensing, drug and gene delivery, and tissue engineering [4].

Several techniques have been reported to produce graphene such as mechanical and electrochemical exfoliation of graphite, chemical vapor deposition, plasma enhanced chemical vapor deposition, thermal decomposition on silicon carbide (SiC), and reduction of graphene oxide (GO) [6]. Mechanical exfoliation of graphite can be either performed through atomic force microscope (AFM) probe techniques or adhesive tape exfoliation and results in high quality graphene. However, the high production cost makes this technique only feasible for research purposes [6,7]. Chemical vapor deposition is the most used technique for large-scale production of single or few-layer graphene [6,8]. Chemical vapor deposition is an expensive process due to the large energy consumption and the necessity of removing the substrate. Besides, controlling the grain size and the number of graphene layers produced is still a challenge [8]. Graphene produced by this technique has high quality when the processing parameters are properly controlled. The main drawbacks are the high cost of SiC wafers and the high temperatures involved in the process (around 1200 °C) [4,8].

Reduction of GO appears to be another viable route to produce single-layer graphene [9]. GO can be prepared from graphite through various methods such as those reported by Brodie, Staudenmeier and Hummers [10], either following their original protocol or introducing some variations. The oxidation process of graphite introduces hydroxyl, epoxy, carbonyl, and carboxyl groups in the hexagonal lattice of graphene [8]. Then, the reduction of GO can be performed, for instance, through a chemical approach to remove the oxygen-containing functional groups and restore the conjugated graphene structure [11]. Compared to others, this process allows the production of large quantities of graphene at a low cost since no special equipment or high temperatures are needed and the starting materials, graphite and chemical reductants, such as hydrazine and sodium borohydride, are usually cost-effective [12].

The major drawback of the traditional routes for chemical reduction of GO is the use of toxic and hazardous chemicals both to living organisms and to the environment [12]. Thus, special care with the handling of these chemicals must be taken and, at an industrial scale, the remediation of the hazardous wastes generated might result in a substantial increase of the production costs. In addition, if toxic residues are still present in the final material, applications in the biological and biomedical fields are unsuitable [13].

Regarding the reduction of graphene oxide, an improvement in the use of green chemistry [14] has been observed and several potential environmental-friendly chemicals have been studied. Among these, vitamins [15,16,17], saccharides [18], amino acids [19,20,21], organic acids [22,23,24,25], microorganisms [26,27], proteins and peptides [28,29], hormones [30], urea [31], and plant extracts [32,33,34,35] have been tested as reducing agents for GO. Dopamine, a nature-based, commercially available, and inexpensive reagent, was first employed for this purpose in 2010 by Xu et al. [36]. Nevertheless, a substantive interpretation of data showing the successful reduction of GO at a molecular lever was not provided. In the presence of the oxygen functional groups in GO and at a weak alkaline pH, dopamine self-polymerizes to form polydopamine (PDA) with the catechol groups undergoing oxidation to form quinone groups. Thus, the polymerization of dopamine at the surface of GO is accompanied by the reduction of the last with dopamine acting both as a reducing and functionalization agent [36]. The presence of PDA on the surface of reduced graphene oxide (RGO) allows the preparation of more stable dispersions, compared with RGO prepared using other reducing agents, which can be a crucial factor for further processing through liquid assisted techniques. Furthermore, PDA strongly adheres to a wide range of substrates, which makes it a good material for applications in functional coatings [37]. Since 2010, several works have been published on the production of PDA-GO envisaging applications such as water purification [38], anion and proton exchange membrane fuel cells [39], functional coatings, and biomedical applications, for instance cancer treatment [40], drug delivery [41], antibacterial materials [42], biosensing [43], and tissue engineering [44]. However, these works focus mainly on the use of PDA-GO as a platform for anchoring of nanoparticles or covalent grafting of other molecules and on the characterization of the materials prepared envisaging the final application and lack deep insight into the mechanism of the GO reduction by PDA.

Considering the limited knowledge on this topic, in this work, we propose a new approach using X-Ray photoelectron spectroscopy (XPS) to supply evidence for the molecular reduction of GO by PDA. This reduction process is a good example of the use of green chemistry to replace traditional methods since no toxic solvents are used and PDA is a natural and renewable raw material, and only a little amount of waste is generated. Our investigation contributes to the field of conductive composite material development based on the strategy to use inexpensive and easily available graphite as basic raw material. In addition, the removal of the PDA to obtain RGO is studied. For this purpose, GO, PDA-GO, and RGO were prepared and characterized by Raman spectroscopy, XPS, thermogravimetric analysis (TGA), and electrical powder conductivity measurements. Thus, the effect of the chemical treatments in the thermal and electrical properties of GO and PDA-GO was studied and a better understanding of the chemistry behind the green reduction of GO with PDA is presented.

## 2. Materials and Methods

### 2.1. Materials

Natural graphite flakes (99%; −325 mesh), tris(hydroxymethyl)aminomethane (tris base, ≥99.8%), dopamine hydrochloride (DA, 98%), and sodium hydroxide (NaOH, >97%) were purchased from Sigma Aldrich^®^ (Munich, Germany). Sulfuric acid (H_2_SO_4_, 98%), potassium permanganate (KMnO_4_, ≥99%), and hydrochloric acid (HCl, 10% v/v) were received from VRW^®^ (Darmstadt, Germany). Hydrogen peroxide (H_2_O_2_, 30% w/v) was purchased from Merck (Darmstadt, Germany). Distilled water (DW) was used in all chemical treatments.

### 2.2. Synthesis of Graphene Oxide, Polydopamine-Reduced Graphene Oxide, and Reduced Graphene Oxide

Graphene oxide (GO) powders were prepared through a modified Hummers’ method according to our previous publication [45]. The oxidation step was performed using H_2_SO_4_ and KMnO_4_ (graphite:KMnO_4_ = 1:1). DW, H_2_O_2_, and HCl were used for the purification step.

For the synthesis of PDA-GO, first a tris base solution in DW (0.1 M) was prepared and degassed by N_2_ bubbling during 20 min. Then, 40 mg of GO were placed in a round bottomed flask and degassed for 10 min with N_2_. After that, 20 mL of tris base were added, and the suspension was magnetically stirred for 15 min at 60 °C. After stirring, DA was added to the GO suspension (GO:DA = 1 w/w) and the suspension was degassed by N_2_ bubbling during 10 min. The reactions between GO and DA took place at 60 °C for the times indicated in Table 1. After the reaction, the suspension was vacuum filtered with a nylon filter membrane (0.45 μm pore size, Whatman, Kent, UK). Finally, the powders were collected and dried overnight in vacuum at 60 °C.

Reduced graphene oxide (RGO) was prepared from PDA-GO. First, 20 mg of PDA-GO_30, PDA-GO_90, and PDA-GO_180 were stirred each in a 40 mL of NaOH solution (5 M), under N_2_ flow for 6 h. Then, the suspension was vacuum filtered with a nylon filter membrane (0.45 μm pore size, Whatman, Kent, UK), and the powders collected and dried at 60 °C under vacuum, overnight, to obtain RGO_30, RGO_90, and RGO_180.

### 2.3. Modeling and Characterization of GO, PDA-GO, and RGO

#### 2.3.1. Ab-Initio Calculations

Ab-initio calculations were performed to get information about an optimized geometry of GO by minimizing the corresponding Hartee–Fock energy applying the software package GAMESS (freeware version 2017-09-30R2, Iowa State University, Ames, IA, USA) [46] using the basis set STO-6G. For this purpose, first, a graphene-like structure consisting of four rows of heptacene was considered. Then, as a first oxidation step, the formation of external hydroxyl and carboxyl groups was simulated following the production of oxirane groups in the plane of the graphene-like molecule. Finally, the oxirane rings opening and conversion into hydroxyl groups, due to the acidic medium where the reaction takes place, was calculated.

#### 2.3.2. Raman Spectroscopy

Raman Spectroscopy was performed on a Raman microscope alpha300R (WITec, Ulm, Germany) using a laser excitation wavelength of 532 nm, laser power of 1 mW, a spectral resolution of 6 cm^−1^, and integration time of 0.5 s. Two hundred scans were accumulated to record each spectrum. For this purpose, GO, PDA-GO, and RGO aqueous suspensions were prepared and sprayed on a glass slide positioned on a heating plate for fast water evaporation and deposition of the graphene products to be analyzed. The *I_D_*/*I_G_* or *I_D_*_+PDA_/*I_G_*_+PDA_ ratios were calculated considering the area under curve of the bands, which were estimated with a mean error of about 10%.

#### 2.3.3. X-Ray Photoelectron Spectroscopy (XPS)

XPS studies were carried out by means of an Axis Ultra photoelectron spectrometer (Kratos Analytical, Manchester, UK). The spectrometer was equipped with a monochromatic Al Kα (hν = 1486.6 eV), X-ray source of 300 W at 15 kV. The kinetic energy of photoelectrons was determined with hemispheric analyzer set to pass energy of 160 eV for wide-scan spectra and 20 eV for high-resolution spectra. For the C 1s region, the maximum information depth of the XPS method is about 8 nm [47,48]. Employing Scotch double-sided adhesive tape (3M Company, Maplewood, MN, USA), the powdery samples were prepared as thick films on a sample holder. During all measurements, electrostatic charging of the sample was avoided by means of a low-energy electron source working in combination with a magnetic immersion lens. Later, all recorded peaks were shifted by the same value that was necessary to set the component peak *Gr* showing the sp²-hybridized carbon atoms of the graphite-like lattice (–C=C– ↔ =C–C=) to 283.99 eV [49]. In the case of considerable amounts of saturated hydrocarbons (PDA-GO samples), their corresponding component peaks in the C 1s spectrum was used as a reference with a binding energy of 285.00 eV [50]. Quantitative elemental compositions were determined from peak areas using experimentally determined sensitivity factors and the spectrometer transmission function. The shapes of the high-resolution element spectra were used to analyze the different binding states of the elements. For this purpose, the high-resolution element spectra were deconvoluted into component peaks (Kratos spectra deconvolution software, software version 2.2.9, Kratos Analytical Ltd., Manchester, UK), in which their binding energy values (BE), height, full width at half maximum and the Gaussian–Lorentzian ratios were free parameters.

#### 2.3.4. Thermogravimetric Analysis (TGA)

TGA measurements were carried out on a TGA Q 5000 (TA Instruments Inc., New Castle, DE, USA) under N_2_ atmosphere between 40 to 800 °C and heating rate of 10 K min^−1^. Prior to the measurements, the powders, except graphite, were dried at 100 °C for 12 min using the same equipment.

#### 2.3.5. Electrical Powder Conductivity

**Figure 1:**
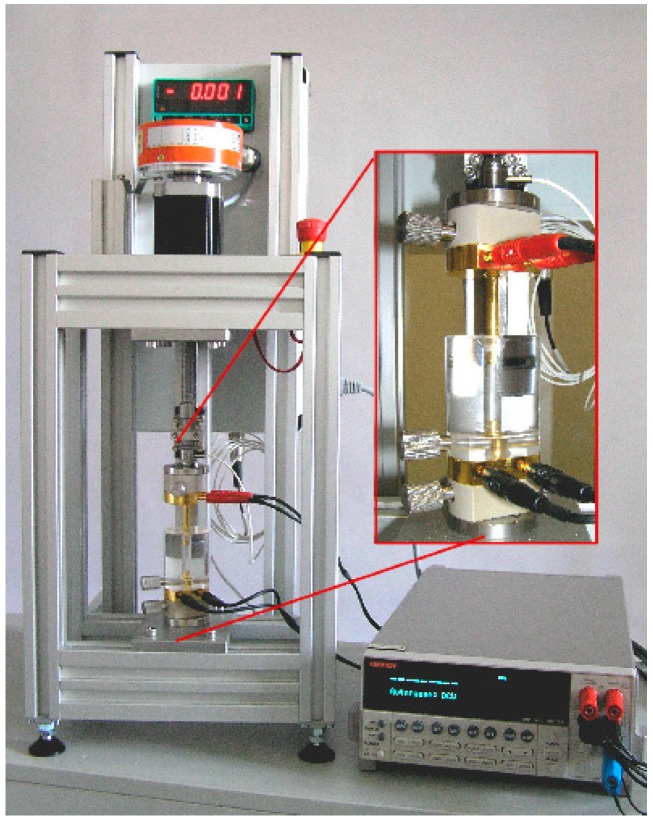
Equipment used for electrical powder conductivity measurements (photo by Dr. Wolfgang Jenschke, Leibniz Institute of Polymer Research Dresden, Dresden, Germany).

The electrical conductivity of the powders was measured using the equipment shown in Figure 1, which was developed and constructed at the Leibniz Institute of Polymer Research Dresden. The equipment consists of a transparent cylinder of 40 mm length with a capillary hole with diameter of 5 mm, which is mounted on a gold electrode on its bottom. After a certain amount of powder (~25 mg) was filled inside the hole, the upper movable cylindrical gold electrode with the same diameter compresses the material stepwise up to a pressure of 30 MPa using a stepper motor. The resistance was measured between the two gold electrodes using a Keithley 2001 electrometer (Tektronix, Köln, Germany) and the conductivity was calculated [51]. At least three measurements were performed to get mean values and standard deviations. In addition, based on the weighed powder mass and the volume of the sample, given by the geometrical conditions of the cylinder and the position of the stepper motor, the bulk density and its development at different pressures can be determined. This also gives a measure of the compressibility of the powder.

## 3. Results and Discussion

### 3.1. The Oxidation of Graphene Stacks

**Figure 2:**
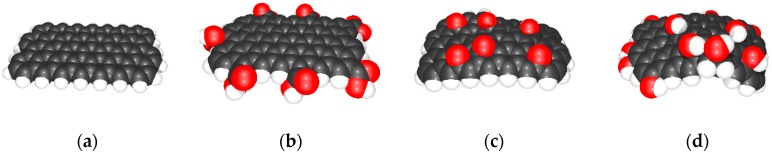
Results of ab-initio calculations to model the geometries of graphene-like and GO-like molecules: (**a**) graphene-like structure consisting of four rows of heptacene; (**b**) oxidized graphene carrying sterically demanding carboxylic groups on the edge; (**c**) oxidative attack on the carbon atoms in the molecular plane formed oxiran groups instable in acidic media; and (**d**) GO-like molecule decorated with hydroxyl groups on the edge and in the molecular plane.

Graphene is an allotrope form of carbon with a 2D honeycomb structure. According to the sp^2^ orbital hybridization of all its carbon atoms, it can be considered as an infinitely large polycyclic aromatic molecule. While the electrons in the s, p_x_ and p_y_ orbitals form σ-bonds, the p_z_ electronsare involved in conjugated π-bonds hybridizing to π-band and π∗-bands. The half-filled π-band covering the whole molecule permits free-moving electrons, which are responsible for the graphene’s electrical conductivity. In fact, the structure of a real graphene sample must be limited. Figure 2a shows a graphene-like model molecule consisting of four rows of heptacene. The degree of oxidation of all these graphene-like carbon atoms can be theoretically given to an average number of −0.307. To understand the oxidation of graphene, in a model based on the Lerf–Klinowski model [52,53], the edges of a graphene sample were decorated with hydrogen atoms to avoid a geometric disorder and keep the aromaticity and electrical conductivity of the molecule [54,55].

As can be seen in Figure 2a, the optimized hydrogen-decorated graphene-like model molecule is flat and planar. With increasing degree of oxidation (exchange of 7 external H atoms with 7 carboxyl groups increases the average oxidation number of carbon atoms to 0.047), the original honeycomb structure was disturbed. At first, external hydroxyl and carboxyl groups (probably also other carbonyl groups, such as quinone-like groups, which were not studied here) slightly deformed the planarity of the graphene-like molecule (Figure 2b). The oxidative attack on carbon atoms in the plane of the graphene-like molecule may produce oxirane groups (Figure 2c). However, due to the strongly acidic medium where the oxidation of graphene took place, the oxiran rings were opened immediately and converted into hydroxyl groups, as shown in Figure 2d. Regardless of whether hydroxyl or oxiran groups were formed in the molecular plane, the former flat plane bended up and needed more space. This effect is often used to separate single layers of graphene from their stacks. The combination of hydroxyl groups with the replacement of edge-standing hydrogens by carboxyl groups increases the average degree of oxidation of carbon atoms to 0.176.

### 3.2. Raman Spectroscopy

**Figure 3:**
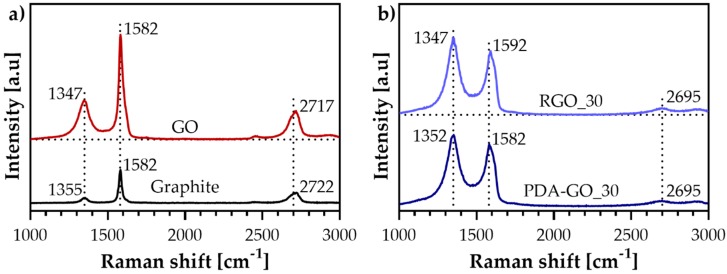

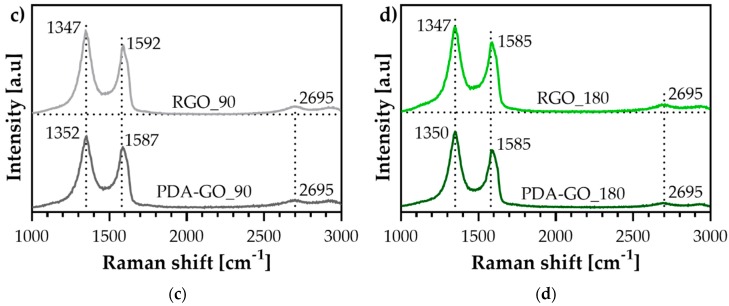
Raman spectra and position of the *D*, *G*, and *2D* bands of: (**a**) graphite and GO; (**b**) PDA-GO_30 and RGO_30; (**c**) PDA-GO_90 and RGO_90; and (**d**) PDA-GO_180 and RGO_180.

Raman Spectroscopy was performed to evaluate the level of “disorder” of the sp^2^ hybridized structure of the materials prepared. The Raman spectra, presented in Figure 3, show the three major bands characteristic of sp^2^ carbon materials. The *D* band, near 1350 cm^−1^, is related to the presence of structural defects in the hexagonal sp^2^ carbon lattice of graphene and to edge effects [56,57]. The *G* band, at approximately 1580 cm^−1^, is related to the in-plane vibration of the sp^2^ carbon atoms [56]. The band near 2700 cm^−1^, the *2D* band, originates on a second-order Raman scattering process and its shape, width, and position is related to the number of layers for *n*-layer graphene. Ferrari et al. reported that an increase in the number of layers originates a broader *2D* band shifted to higher Raman shifts. [57,58]. The relative signal intensity of the *D* band to the *G* band (*I_D_*/*I_G_*) provides information about the level of “disorder” in terms of covalent modification of the graphene structure [57,59]. In pristine graphite, the *2D* band consists of two components and appears at ≈2720 cm^−1^ while graphene presents a single sharp peak centered at a Raman shift lower than 2700 cm^−1^ [59].

Regarding PDA, there are two bands at about 1358 and 1588 cm^−1^ [45] that are assigned to the stretching vibration and deformation of chatecol groups [60]. Thus, the *D* and *G* typical for the graphene derivatives overlap with the PDA and a proper assignment of these bands is not possible. As long as PDA is present at the GO surface, contributions of these bands have to be considered. Thus, particularly the band at 1350–1358 cm^−1^ presents an overlap of the signals coming from the structural defects of GO, functional groups on the surface, and the amount of PDA in the material. Nevertheless, the ratios between the intensities at about 1350 and 1580 cm^−1^ were calculated and referred to as *I*_*D*+PDA_/*I*_*G*+PDA_ for the PDA containing samples.

The Raman spectra in Figure 3a show an increase of the *I_D_*/*I_G_* ratio when graphite was chemically converted in GO, from 0.2 to 0.5. This is a consequence of an increase of the content of structural defects caused by the introduction of oxygen-containing functional groups during the oxidation process or by the decrease in the flake size when GO was exposed to sonication [61]. Moreover, the downshift to 2717 cm^−1^ after oxidation indicates a reduction in the number of graphene stacks.

After reduction with PDA, a further increase in *I*_*D*+PDA_/*I*_*G*+PDA_ was observed, as shown in Table 2. Raman spectroscopy is a standard method to evaluate the molecular structure of graphene and graphene derivatives. However, considering the overlapping bands of GO and PDA, in this case, the method is not well suited to determine if the reduction and restoration of the sp^2^ structure occurred since the typical *I_D_*/*I_G_* ratios also have a contribution of the PDA signal. Nevertheless, the position of the *2D* band shows a further reduction on the number of graphene stacks on the PDA-GO materials.

The evaluation of the structure of RGO should be possible since no contribution from PDA peaks is expected. However, no significant changes were found between the spectra of RGO and the corresponding PDA-GO, which might indicate that the PDA was not completely removed after the treatment with NaOH. Thus, additional experiments are required to study the molecular structure of these materials by Raman Spectroscopy and to optimize the removal of the PDA layer. In addition, different methods, such as XPS, are required to properly characterize the molecular structure of PDA-GO.

### 3.3. X-Ray Photoelectron Spectroscopy (XPS)

#### 3.3.1. XPS Spectra of Graphene Stacks and GO

**Figure 4:**
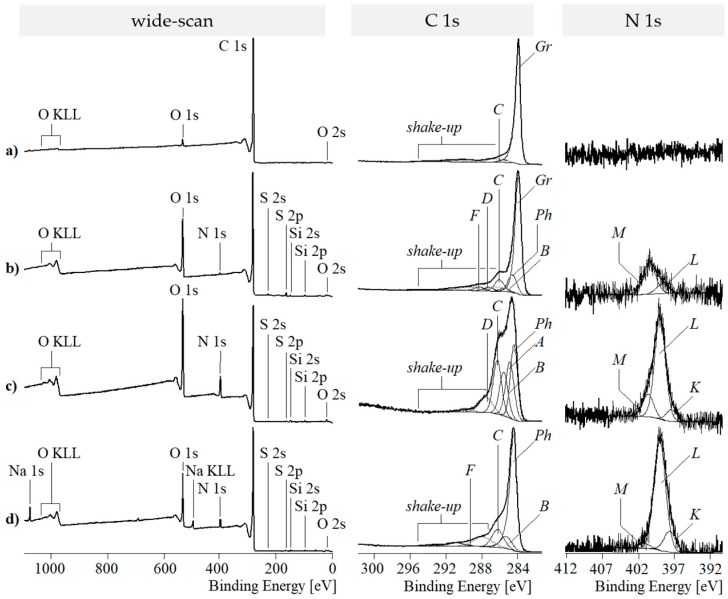
Wide-scan (left column), high-resolution C 1s (middle column) and N 1s (right column) XPS spectra recorded from: (**a**) graphene stacks; (**b**) GO; (**c**) PDA-GO_30; and (**d**) RGO_30.

The XPS studies corroborate the information obtained by Raman spectroscopy. The shape of the C 1s spectrum of graphite in Figure 4a is very characteristic for carbonaceous substances consisting of sp^2^ hybridized lattices. Due to the numerous excited states, the spectrum noticeably tailed on the high energy side. The main component peak *Gr* found at 283.99 eV results from photoelectrons that escaped from sp^2^-hybridized carbon atoms (–C=C– ↔ =C–C=). Photoelectrons removed from molecules with exited electron states appear as wide shake-up peaks (gray lines in Figure 4). At 285.69 eV, a further small component peak *C* was observed. This component peak shows the presence of C–O bonds on the sample surface (detailed XPS data are presented in Tables S1–S18 of the Supplementary Materials). 

The oxidation of graphite to GO significantly increases the relative oxygen content from 0.028 to 0.166 (Figure 4b). This increase results from the presence of numerous oxygen-containing functional groups on the sample surface. However, the C 1s spectrum (Figure 4b, middle column) shows that the oxidation reaction was gently performed. It seemed to be beneficial to minimize the destruction of the conjugated π-electron system by breaking σ-bonds during the formation of carboxylic acid groups. On the other hand, the planar graphene sheets should be sufficiently oxidized to bend them and expand the former stacks. The predominant preservation of the π-electron system becomes clear by the appearance of the intense component peak *Gr* and the shake-up peaks. It also seems necessary to introduce a component peak *Ph* (284.75 eV), indicating the presence of sp^2^-hybridized carbon atoms that are not involved in the highly conjugated π-system of the original graphene lattice [50]. Oxygen-containing functional groups were analyzed as component peaks *C* (286.07 eV), *D* (287.29 eV), and *F* (288.36 eV). Component peak *C* results from photoelectrons that escaped from phenolic C–OH groups. The binding energy value found for component peak *C* is also characteristic for ether groups (–C–O–C–). However, as mentioned above, it is unlikely that oxirane groups are stable on the graphene surface. Component peak *D* shows carbonyl carbon atoms of quinone-like (C=O) groups. Photoelectrons from the carbonyl carbon atoms of carboxylic acid groups (O=C–OH) and their corresponding carboxylates (O=C–O^Ө^ ↔ ^Ө^O–C=O) were observed as the small component peak *F*. The presence of nitrogen ([N]:[C] = 0.009) required the introduction of an additional small component peak *B* (at 285.72 eV) showing C–N bonds. These bonds can be constituents of amino and/or amide groups. In the case of the presence of amide groups, photoelectrons of the carbonyl carbon atoms (O=C–NH–C) contributes to component peak *D* while component peak *B* presents the amine-sided carbon atoms (O=C–NH–C).

#### 3.3.2. XPS Spectra of PDA-GO

**Figure 5:**
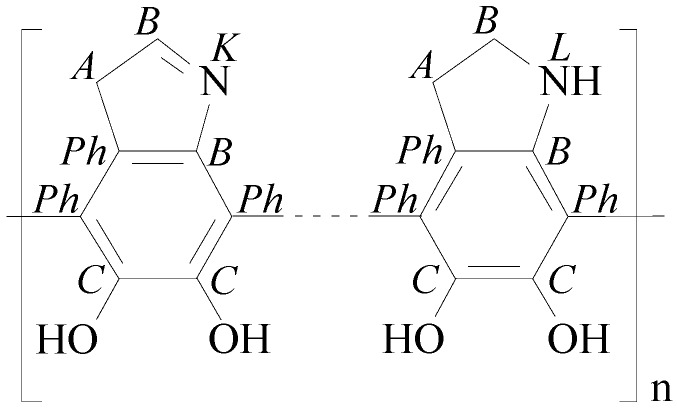
Characteristic cutouts from the chemical structure of PDA. Italic letters denote the assignment of the carbon and nitrogen atoms to the component peaks in the C 1s and N 1s high-resolution spectra.

The application of dopamine and its subsequent polymerization on the GO surface significantly increased the relative nitrogen content from [N]:[C] of the samples from 0.009 to ca. 0.090. The variation of the time for the polymerization reaction from 30 to 210 min does not correlate with the relative nitrogen content on the GO surfaces and thus with the amount of PDA deposited there. As can be seen in Figure 4c, the PDA layer strongly changes the shape of the C 1s spectra. Carbon atoms bonded in the phenyl rings of the PDA’s catechol groups having no heteroatoms as binding partner and sp²-hybridized carbon atoms from the graphite-like lattice of the substrate materials were identified as component peak *Ph* (ca. 284.49 eV). Component peak *A* (285.00 eV) results from the presence of carbon atoms in the sp³-hybrid state of saturated hydrocarbons. Some of these carbon atoms are constituents of the PDA layer, but the presence of component peak *A* can be also considered as a first hint of reduced carbon species on the sample surface. Carbon–nitrogen bonds were assigned as component peak *B* (ca. 285.72 eV). The intensities of the component peaks *B* equal the twice of the [N]:[C] ratios determined from the corresponding wide-scan spectra. According to the structural formula of the PDA in Figure 5, the number of carbon atoms carrying phenolic OH groups (C–OH) should equal the number of carbon atoms bonded to nitrogen.

**Figure 6:**
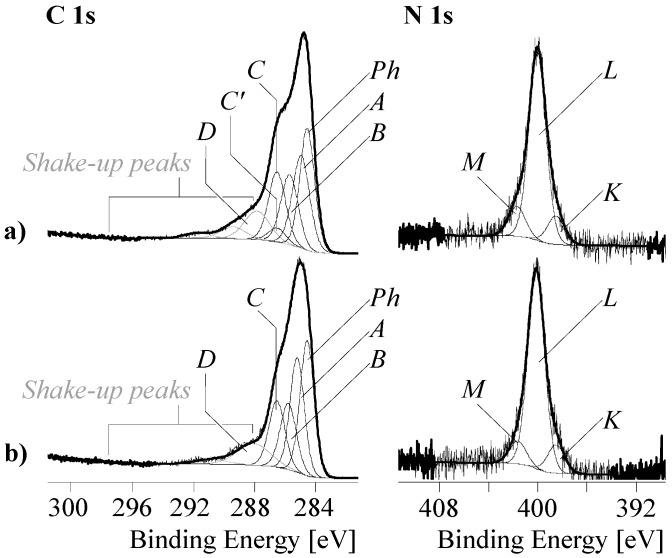
High-resolution C 1s (left) and N 1s (right) XPS spectra recorded from: (**a**) PDA-GO_90; and (**b**) PDA-GO_180.

However, the C 1s spectra recorded from the samples that reacted for 30 and 90 min showed component peaks *C* with higher intensities than their corresponding component peaks *B*, as shown in Figure 6a. It is assumed that the excess ([*C’*] = [*C*] − [*B*]) of the intensities of the component peaks *C* result from the contribution of C–OH groups from the GO substrate material. Quinone-like groups cannot be safely detected because component peak *D* is overlapped by intense shake-up peaks.

After longer polymerization times, such as 180 and 210 min, the excess component peaks *C’* disappears completely (Figure 6b). Obviously, the reduction of the GO by the oxidative polymerization of the adsorbed dopamine molecules requires longer periods of reaction time. Figure 6b (right column) shows that the high-resolution N 1s spectra recorded from the PDA-GO samples is deconvoluted into the three component peaks *K* (ca. 398.69 eV), *L* (ca. 400.1 eV) and *M* (ca. 401.68 eV). Corresponding to the intensities of the component peaks *B* (in the C 1s spectra), which are the twice of the [N]:[C] ratios and the binding energy values found, component peak *L* shows cyclic secondary amino groups (C–NH–C), such as the pyrrolidine structures in Figure 5 on the right side [50,62]. The binding energy values of the component peaks *K* are unusually small for organically bonded nitrogen. The observation of such low binding energy values indicates a high electron density at the nitrogen atom, which is characteristic for the cyclic imide nitrogen atoms (C–N=C) exemplary shown in Figure 5 (left). Protonated nitrogen species (C–N^+^H) reflecting the protonation/deprotonation equilibrium of Brønsted basic nitrogen species are observed as component peak *M*.

#### 3.3.3. XPS Spectra of RGO

NaOH was employed to hydrolyze and remove the PDA layer covering the carbonaceous substrate materials. However, the wide-scan spectra shows the presence of considerable amounts of nitrogen ([N]:[C] ≈ 0.04) after treating the samples with NaOH (Figure 4d, left column). These findings can be considered as a hint that PDA was not completely removed from the substrate materials. Nevertheless, the corresponding C 1s spectra in Figure 4d exemplary shows the C 1s spectrum of sample RGO_30 with clearly intensity-reduced shoulders in the region of 286.5 eV and tailings on the high energy sides, which were previously observed in the C 1s spectrum of the graphene reference sample (Figure 4a, middle column). Corresponding to the findings of the PDA-coated samples, component peak *B* (285.34 eV) appears with an intensity that is twice the [N]:[C] ratio found in the wide-scan spectrum of sample RGO_30. An equal number of photoelectrons escapes from the PDA’s catecholic C–OH groups and contribute to component peak *C* at 286.29 eV. The excess of component peak *C* ([*C’*] = 0.061) has to be assigned to the C–O groups remaining on the surface of the substrate material. Probably, component peak *D* disappears completely while small traces of carboxylic acid groups (O=C–OH) and/or carboxylic ester groups (O=C–O–C) were observed as component peak *F* (289.36 eV). Latter groups could be formed by esterification of the carboxylic acid groups with some of the PDA’s catechol groups. Photoelectrons of the corresponding alcohol-sited carbon atoms (O=C–O–C) contribute to component peak *C*. As discussed above, the N 1s spectrum was deconvoluted into the three component peaks *K*, *L* and *M*.

In summary, in the case of organic materials, it is often very difficult to determine or estimate the contribution of surface contaminations to the spectra. This is especially true for very complex-shaped high-resolution spectra, which were recorded for the samples studied here. C 1s spectra recorded from the graphene stacks, GO, and RGO_30 provide no information about the degrees of contaminations. A component peak showing the presence of surface contaminations arise at the same peak position. A separation was not possible. If they are present and can be separated [63] the photoelectrons of their carbon atoms would contribute to component peak observable at about 285 eV.

As shown in Figure 4, we found slightly different binding energy values for the component peaks *C*. The reason of these differences was the different chemical character of the C–OH groups. In the case of the PDA-GO samples (PDA-GO_30, PDA-GO_90, and PDA-GO_180), C–OH groups of the di-phenolic catechol units mainly contributed to the component peaks *C* (about 286.47 eV). After their removal with NaOH (sample RGO_30), a few residual di-phenolic catechol units and numerous OH groups from the not fully reduced substrate material contributed to component peak *C*. The binding energy value was slightly lowered (286.29 eV). The phenolic OH groups on the GO, which were more intensively involved in the delocalized p-electron system of the (more or less disturbed by oxidation) graphite-like lattice, showed a binding energy value of 286.07 eV. The component peak *C* resulting from photoelectrons of traces of oxygen-carrying functional groups found on the surfaces of the graphene stacks had a binding energy value of 285.69 eV, which is significantly lower as usually expected for C–OH bonds. However, here we have to take into account that these few phenol groups are embedded in a largely undisturbed p-system, and - in contrast to the catechol rings - their dissociations (C–OH + H_2_O to C–O^−^ + H_3_O^+^) increasing the electron density at the carbon atoms are not hindered by negative charges in their immediate molecular neighborhood.

In all cases, the shapes of the component peaks were the result of convolutions of a Gaussian normal distribution and Cauchy–Lorentz distribution. With exception of the distribution of the component peak *Gr* and the component peak *Ph* in the RGO_30 sample, all other component peaks had the same shapes as suggested by the reviewer. High number of excited states in the well-ordered graphite-like lattice led to a tailing of the photoelectron distribution at the high-energy side of the component peak. Hence, it seemed to be necessary to adapt the line shape of the component peaks *Gr* by an increased asymmetry. In the case of the component peaks *Ph* in sample RGO_30, we have to consider that this component peak *Ph* summarized photoelectrons from the p-conjugated carbon atoms of the remaining PDA molecules and the carbon atoms of the more or less disturbed graphite-like structures of the substrate.

Furthermore, the XPS spectra recorded from the PDA-GO samples clearly show the reduction of the GO. As can be seen in the high-resolution C 1s spectrum, the reduction is mainly due to the decrease in the C–OH groups of the former GO substrate. The majority of the C–OH groups found in that spectrum are constituents of the PDA, which remain on the sample surface after washing with NaOH. The PDA wrapping the carbonaceous material can be used as stable anchor layer for subsequent modification reactions to functionalize and compatibilize carbon-based nanomaterials.

### 3.4. Thermogravimetric Analysis (TGA)

**Figure 7:**
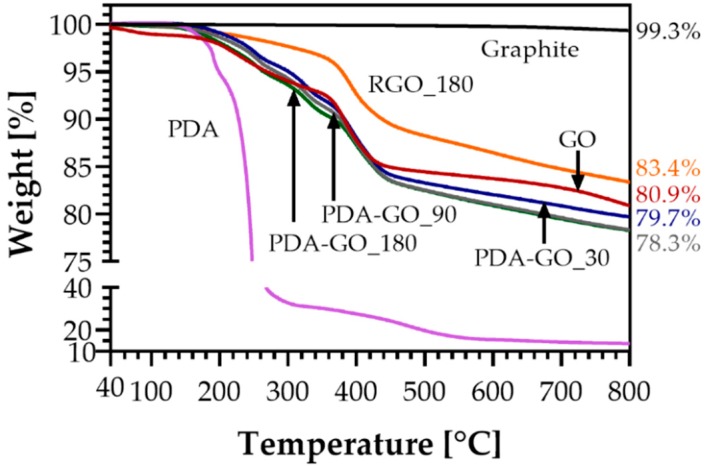
Thermogravimetric analysis (TGA) curves of graphite, GO, PDA, PDA-GO_30, PDA-GO_90, PDA-GO_180, and RGO_180.

**Figure 8:**
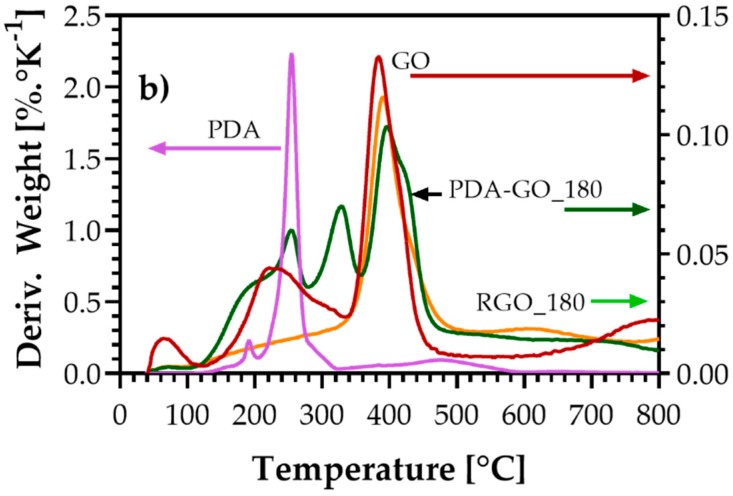
Derivative weight loss of GO, PDA, PDA-GO_180, and RGO_180.

TGA was performed to evaluate the thermal stability of graphite, GO, PDA-GO, and RGO. It was expected that the amount of PDA on the GO surfaces could be evaluated from the TGA results. As shown in Figure 7, PDA has the lowest thermal stability, starting thermal degradation around 180 °C due to the partial decomposition of the main chain in three different stages with a maximum at around 260 °C [64]. The weight loss observed for graphite is very low and mainly due to the release of adsorbed water and residual oxidation [61]. Regarding GO, Figure 8 shows three loss stages. The first weight loss stage of ca. 1.7% occurring between 40 and 120 °C was attributed to the evaporation of adsorbed water [65]. The second weight loss (5.7%) has its maximum at about 220 °C and might be due to the decomposition of oxygen-containing functional groups (C–OH), whereas the main weight loss (8.8%) occurs at about 400 °C and is related to the loss of more stable oxygen functionalities such as carboxyl groups. The total weight losses of graphite, GO, and PDA at 800 °C are 0.7%, 19.1%, and 86.3%, respectively.

The TGA curves of the PDA-GO materials show significant thermal degradation starting at around 180 °C due to the decomposition of PDA and the removal of non-reduced oxygen-containing functional groups from GO. The different polymerization times only slightly affected the thermal stability of the PDA-GO materials, which underwent a total weight loss of between 20.3% (PDA-GO_30) and 21.7% (PDA-GO_90 and PDA-GO_180) at 800 °C, as shown in Figure 7. However, a decrease in the thermal stability in comparison with GO was observed, possibly related to the increase of the oxygen content as determined by XPS due to the presence of PDA. Regarding RGO_180, the curve in Figure 8 shows only a weight loss stage with a maximum at around 400 °C, relative to the decomposition of stable oxygen-containing functional groups which were not reduced by PDA as determined by XPS. No peaks from PDA contribution were found throughout the temperature range analyzed; however, the small weight loss that occurs below 300 °C might be attributed to the decomposition of the residual PDA remaining after NaOH treatment. A total weight loss of 16.6% was observed, which reflects an improvement of the thermal stability after PDA removal. As calculated by XPS, RGO_180 possesses the lowest amount of oxygen species. which is reflected by its highest thermal stability among all prepared materials.

### 3.5. Electrical Powder Conductivity

**Figure 9:**
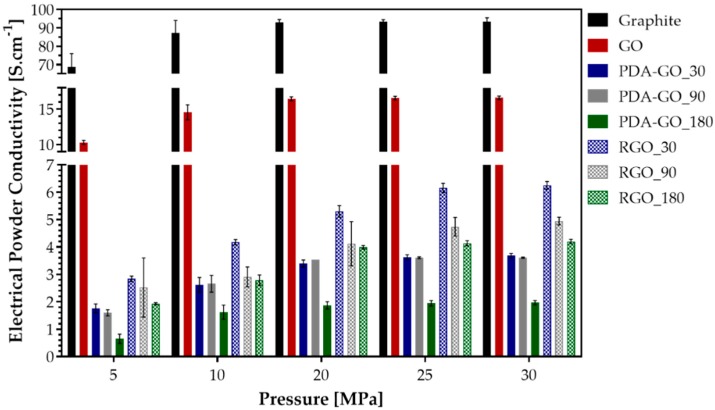
Electrical conductivity of the powdery materials at different pressures.

The electrical powder conductivity of the graphene derivatives was measured to evaluate the effect of the reduction process on the restoration of the electrical properties. The electrical powder conductivity generally increases with the pressure applied during the measurements. As shown in Figure 9, the starting material graphite has the highest electrical conductivity among all materials with values between 68.8 and 93.4 S/cm. As expected, after oxidation, there was a remarkable decrease in the conductivity of about 18% to 16.6 S/cm (at 30 MPa). This value of GO is higher than values reported previously when using the same measurement equipment but differently oxidized GO materials [66,67]. The reduction of GO with PDA resulted in a substantial decrease in the electrical powder conductivity, which scales with the reaction time between GO and DA and is most significant in PDA-GO_180 with a decrease to 1.9 S/cm at 30 MPa. Although the reduction process allowed the removal of oxygen-containing functionalities and potentially a restoration of the sp^2^-hybridized lattice of graphene, the electrical insulating nature of the PDA layer might contribute to this effect. By this, a decrease in the contact area between conductive graphene areas and an increase in contact resistance between the partially PDA covered conductive graphene occur, which justifies the decrease in the electrical conductivity. The conductivity of PDA-GO_180 increases with the pressure until 20 MPa, remaining constant afterwards. Regarding PDA-GO_30 and PDA-GO_90, similar electrical powder conductivity values were measured for all pressures considered. For PDA-GO_30, there was an increase of 48% from 1.8 to 3.7 S/cm while for PDA-GO_90 an increase of 44% from 1.6 to 3.6 S/cm was observed when increasing the pressure from 5 to 30 MPa.

**Figure 10:**
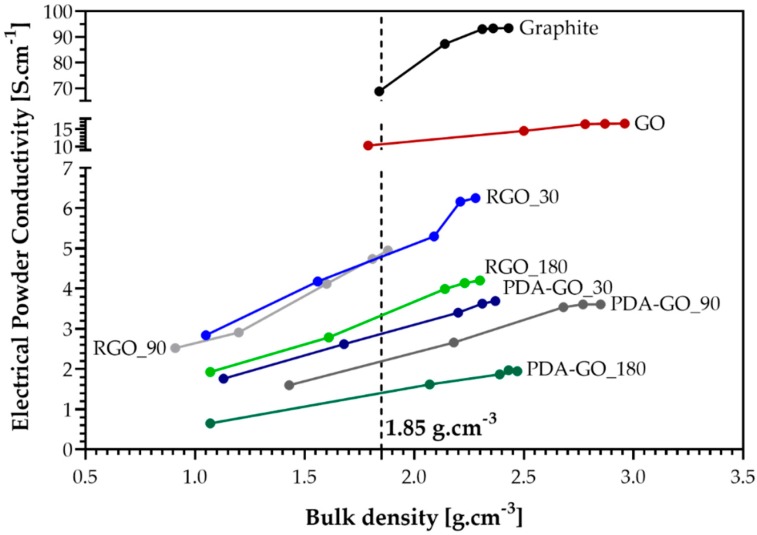
Dependency of electrical conductivity of the powdery materials on bulk density (density increases with pressure).

After the removal of PDA with NaOH, the electrical conductivity increases. When the PDA layer is removed, leaving only a small residue, the resultant RGO possesses a structure more suited for electrical conduction. Following the trend of decreasing conductivity with reaction time between GO and PDA, the samples after PDA removal also show this dependency. However, despite RGO_180 has the lowest conductivity among the RGO samples, the difference between PDA-GO and RGO is most pronounced in this sample which shows an increase of two times after the treatment with NaOH. The values achieved are higher than those reported for differently reduced graphite oxides measured using the same equipment in Ref. [66]. However, even after reduction and PDA removal, the desired higher electrical conductivity than that of GO was not obtained, possibly due to the presence of residual PDA, a more defected sp^2^ carbon structure, or different compressibility of the powders. The representation of the electrical powder conductivity as a function of the bulk density of the various materials in Figure 10 confirms the significant differences between them. Graphite and GO have the highest bulk density values at the applied pressures, which illustrates a higher packing density and better contact between the graphene flakes inside the powder materials. Graphite has a much higher compressibility (especially at pressures between 5 and 20 MPa) than GO, which has the lowest increase in conductivity with pressure, showing the lowest compressibility of all samples. The PDA_GO and RGO powders show lower bulk densities at the initial pressure of 5 MPa and stronger density changes at increasing pressure than graphite and GO. The increase in conductivity with pressure is slightly lower for the PDA_GO than for the RGO samples, demonstrating a slightly higher compressibility of the RGO. This may be a hint that the powder particles in the RGO samples are more flexible and less stiff. The dependencies in Figure 10 illustrate that the materials are in different packing states during the measurements, which affects the contact surfaces and the contact resistance between the material powder particles and flakes in the measured sample volume. If only very small amounts of PDA remain on the surface of graphene flakes, especially at locations where the flakes are in close contact during measurement, this may result in high contact resistance and reduced conductivity through the sample. The comparison of the electrical powder conductivity of all materials at the same density, selected here at 1.85 g cm^−3^, however, confirms the sequence described above at different measuring pressures. Graphite has the highest conductivity, followed by GO, while PDA_GO has a lower conductivity than RGO.

Nevertheless, despite the lower electrical powder conductivity, both PDA-GO and RGO materials possess higher conductivity values than reported for other examples in the literature [66] and show a better dispersibility in several solvents than GO, which is crucial when, for example, these materials are to be used in solvent assisted techniques to prepare conductive films.

## 5. Conclusions

In this work, we propose a new approach to investigate the molecular reduction state of GO by PDA and the removal of the PDA, using NaOH, to obtain RGO. It was shown that Raman spectroscopy is not well suited to determine the reduction and restoration of the sp^2^ structure. However, a first hint for the presence of PDA, even after the treatment with NaOH, can be obtained using this method since no significant differences were found between the PDA-GO and RGO spectra. The reduction of GO by PDA was proven by XPS through a new approach that considers the number of carbon atoms bonded to OH and to nitrogen in PDA and compares it to the total intensity of the signal resulting from OH groups in PDA-GO to finally determine that the reduction occurs. In addition, it was shown that there was no complete removal of the PDA layer with NaOH, corroborating the Raman spectroscopy results. Regarding the thermal analysis, it was observed that the presence of PDA in PDA-GO results in a decrease in the thermal stability. However, after PDA removal, the thermal stability improved and revealed to be higher than in GO, which agrees with the XPS studies that showed RGO_180 possesses the lowest amount of unstable oxygen-containing species. In addition, the graphene derivatives prepared in the present work possess considerably higher electrical powder conductivity values than those reported in the literature, even if the desired higher electrical conductivity than that of GO was not obtained. The small proportion of PDA remaining on the material surface can be used for subsequent functionalization, which often plays the key role for the intended application.

To summarize, in the present work, deep insight into the chemistry of PDA-GO and RGO was given. The green reduction of GO by PDA proved to be a way to replace typical reduction methods that involve toxic, corrosive, and hazardous solvents and chemicals. In the future, further studies are needed to better understand the reduction of GO by such green approaches and make the processes reproducible and scalable.

## Figures and Tables

**Table 1:** Time of reaction between dopamine hydrochloride (DA) and graphene oxide (GO) and respective sample description.

Time of Reaction (min)	Sample Description (PDA-GO_reduction Time in min)
30	PDA-GO_30
60	PDA-GO_60
90	PDA-GO_90
120	PDA-GO_120
150	PDA-GO_150
180	PDA-GO_180
210	PDA-GO_210

**Table 2:** *I_D_*/*I_G_* or *I*_*D*+PDA_/*I*_*G*+PDA_ of the graphene derivatives.

Material	*I_D_*/*I_G_*	*I*_*D*+PDA_/*I*_*G*+PDA_
Graphite	0.2	—
GO	0.5	—
PDA-GO_30	—	1.2
PDA-GO_90	—	1.2
PDA-GO_180	—	1.2
RGO_30	—	1.2
RGO_90	—	1.2
RGO_180	—	1.2

## Supplementary Materials

The following are available online at https://www.mdpi.com/2079-4991/9/6/902/s1, Table S1: Elemental compositions investigated by XPS, Table S2: Wide-scan spectrum of the sample recorded from the graphene stacks, Table S3: C 1s spectrum of the sample recorded from the graphene stacks, Table S4: Wide-scan spectrum of the GO sample, Table S5: C 1s spectrum of the GO sample, Table S6: N 1s spectrum of the GO sample, Table S7: Wide-scan spectrum of the PDA-GO_30 sample, Table S8: C 1s spectrum of the PDA-GO_30 sample, Table S9: N 1s spectrum of the PDA-GO_30 sample, Table S10: Wide-scan spectrum of the RGO_30 sample, Table S11: C 1s spectrum of the RGO_30 sample, Table S12: N 1s spectrum of the RGO_30 sample, Table S13: Wide-scan spectrum of the PDA-GO_90 sample, Table S14: C 1s spectrum of the PDA-GO_90 sample, Table S15: N 1s spectrum of the PDA-GO_90 sample, Table S16: Wide-scan spectrum of the PDA-GO_180 sample, Table S17: C 1s spectrum of the PDA-GO_180 sample, Table S18: N 1s spectrum of the PDA-GO_180 sample.
