# Supplementary material for: Elucidating the Chemistry behind the Reduction of Graphene Oxide Using a Green Approach with Polydopamine
Source: Nanomaterials (Basel). 2019 Jun 21;9(6):902. doi: 10.3390/nano9060902 (PMC6630331; doi:10.3390/nano9060902)
Supplement: Supplementary file 1 [file nanomaterials-09-00902-s001.pdf]

Table S1. Elemental compositions investigated by XPS.

	Graphene stacks	GO	PDA-GO_30	RGO_30
[N]:[C]$\mid_{spec}$	–	0.009	0.096	0.035
[O]:[C]$\mid_{spec}$	0.029	0.166	0.27	0.165
[F]:[C]$\mid_{spec}$	–	–	–	0.005
[Na]:[C]$\mid_{spec}$	0.001	–	0.0004	0.013
[Si]:[C]$\mid_{spec}$	0.004	0.006	0.013	0.005
[S]:[C]$\mid_{spec}$	–	0.012	0.002	0.002
[Cl]:[C]$\mid_{spec}$	0.0003	0.0002	–	0.0002

	PDA-GO_90	PDA-GO_180
[N]:[C]$\mid_{spec}$	0.0831	0.0843
[O]:[C]$\mid_{spec}$	0.2376	0.261
[F]:[C]$\mid_{spec}$	0.003	0.012
[Na]:[C]$\mid_{spec}$	–	–
[Si]:[C]$\mid_{spec}$	0.006	0.024

Table S2. Wide-scan spectrum of the sample recorded from the graphene stacks.

Peak	Position BE (eV)	FWHM (eV)	Raw Area (CPS)	RSF	Atomic Conc %	Mass Conc %
C 1s	284.0	0.58	64387	0.278	96.71	95.17
O 1s	532.7	2.65	5528	0.780	2.76	3.62
Na 1s	1070.6	2. 50	9106	1.685	0.09	0.18
Si 2p	103.2	4.06	6433	0.328	0.41	0.94
Cl 2p	199.3	0.19	1377	0.891	0.03	0.09

Table S3. C 1s spectrum of the sample recorded from the graphene stacks.

Peak	Position BE (eV)	FWHM (eV)	Raw Area (CPS)	Height	Atomic Conc %	Mass Conc %
C 1s *Gr*	284.00	0.75	51530	71650	80.05	80.05
C 1s *C*	285.70	1.00	1840	1675	2.86	2.86
C 1s *Sh1*	286.60	2.34	5302	2067	8.23	8.23
C 1s *Sh2*	289.81	2.34	3389	1321	5.26	5.26
C 1s *Sh3*	291.77	2.34	1933	753	3.00	3.00
C 1s *Sh4*	294.36	2.34	396.0	154	0.61	0.61

C 1s *Gr*: AS(90,0.45).

Table S4. Wide-scan spectrum of the GO sample.

Peak	Position BE (eV)	FWHM (eV)	Raw Area (CPS)	RSF	Atomic Conc %	Mass Conc %
C 1s	284,0	0.90	44545	0.278	83.79	78.23
N 1s	401.2	0.55	731	0.477	0.79	0.86
O 1s	531.6	2.80	22345	0.780	13.90	17.29
Si 2p	101.2	2.45	6503	0.328	0.51	1.12
S 2p	168.2	2.93	25088	0.668	0.98	2.44
Cl 2p	199.3	0.20	855	0.891	0.02	0.07

Table S5. C 1s spectrum of the GO sample.

Peak	Position BE (eV)	FWHM (eV)	Raw Area (CPS)	Height	Atomic Conc %	Mass Conc %
C 1s *Gr*	283.99	0.91	25608	27376	57.50	57.50
C 1s *Ph*	284.75	1.14	5083	4072	11.41	11.41
C 1s *B*	285.72	1.14	808	647	1.81	1.81
C 1s *C*	286.07	1.20	3498	2656	7.85	7.85
C 1s *D*	287.29	1.20	1144	869	2.57	2.57
C 1s *F*	288.36	1.20	1208	916	2.71	2.71
C 1s *Sh1*	286.36	2.36	3461	1335	7.77	7.77
C 1s *Sh2*	289.57	2.36	2212	853	4.96	4.96
C 1s *Sh3*	291.54	2.36	1262	487	2.83	2.83
C 1s *Sh4*	294.13	2.36	261	101	0.58	0.58

C 1s *Gr*: AS(60,1.0).

Table S6. N 1s spectrum of the GO sample.

Peak	Position BE (eV)	FWHM (eV)	Raw Area (CPS)	Height	Atomic Conc %	Mass Conc %
N 1s *L*	399.46	1.83	191	95	26.11	26.11
N 1s *M*	401.15	1.83	540	268	73.89	73.89

Table S7. Wide-scan spectrum of the PDA-GO_30 sample.

Peak	Position BE (eV)	FWHM (eV)	Raw Area (CPS)	RSF	Atomic Conc %	Mass Conc %
C 1s	284.7	2.54	13568	0.278	72.31	66.18
N 1s	400.1	1.74	2299	0.477	6.95	7.42
O 1s	532.6	2.39	11051	0.780	19.57	23.86
Na 1s	1070.9	1.98	815	1.685	0.03	0.05
Si 2p	102.2	2.49	4280	0.328	0.96	2.06
S 2p	168.5	2.07	1584	0.668	0.18	0.43

Table S8. C 1s spectrum of the PDA-GO_30 sample.

Peak	Position BE (eV)	FWHM (eV)	Raw Area (CPS)	Height	Atomic Conc %	Mass Conc %
C 1s *Ph*	284.40	1.08	3729	3474	27.56	27.56
C 1s *A*	285.00	1.05	3022	2623	22.33	22.33
C 1s *B*	285.81	1.13	2600	2097	19.21	19.21
C 1s *C*	286.57	1.13	2600	2097	19.21	19.21
C 1s *C1*	286.57	1.13	0	0	0.00	0.00
C 1s *Sh1*	288.15	1.82	1327	666	9.80	9.80
C 1s *Sh2*	290.80	1.82	256	129	1.89	1.89

Table S9. N 1s spectrum of the PDA-GO_30 sample.

Peak	Position BE (eV)	FWHM (eV)	Raw Area (CPS)	Height	Atomic Conc %	Mass Conc %
N 1s *K*	398.61	1.60	247	141	10.75	10.75
N 1s *L*	400.06	1.60	1680	962	73.13	73.13
N 1s *M*	401.71	1.60	371	212	16.12	16.12

Table S10. Wide-scan spectrum of the RGO_30 sample.

Peak	Position BE (eV)	FWHM (eV)	Raw Area (CPS)	RSF	Atomic Conc %	Mass Conc %
C 1s	284.5	1.00	39897	0.278	81.66	76.34
N 1s	400.0	1.81	2430	0.477	2.82	3.08
O 1s	532.6	3.27	19847	0.780	13.48	16.79
F 1s	689.3	2.91	15673	1.000	0.39	0.58
Na 1s	1070.3	2.44	76371	1.685	1.08	1.94
Si 2p	102.1	2.34	4260	0.328	0.37	0.80
S 2p	168.7	3.29	3880	0.668	0.17	0.41
Cl 2p	199.3	0.59	622	0.891	0.02	0.05

Table S11. C 1s spectrum of the RGO_30 sample.

Peak	Position BE (eV)	FWHM (eV)	Raw Area (CPS)	Height	Atomic Conc %	Mass Conc %
C 1s *Ph*	284.46	1.13	24831	22184	62.03	62.03
C 1s *B*	285.34	1.46	2760	1720	6.89	6.89
C 1s *C*	286.29	1.46	2760	1720	6.89	6.89
C 1s *C′*	286.29	1.46	2441	1521	6.10	6.10
C 1s *F*	289.36	1.46	225	140	0.56	0.56
C 1s *Sh1*	287.92	2.44	4733	1769	11.81	11.81
C 1s *Sh2*	290.77	2.44	1812	677	4.52	4.52
C 1s *Sh3*	293.23	2.44	478	179	1.19	1.19

C 1s *Ph*: AS(80,0.55).

Table S12. N 1s spectrum of the RGO_30 sample.

Peak	Position BE (eV)	FWHM (eV)	Raw Area (CPS)	Height	Atomic Conc %	Mass Conc %
N 1s *K*	398.85	2.09	373	162	15.73	15.73
N 1s *L*	400.07	2.09	1999	964	84.27	84.27

Table S13. Wide-scan spectrum of the PDA-GO_90 sample.

Peak.	Position BE (eV)	FWHM (eV)	Raw Area (CPS)	RSF	Atomic Conc %	Mass Conc %
C 1s	284.8	2.38	12829	0.278	75.21	69.84
N 1s	400.1	1.67	1878	0.477	6.25	6.76
O 1s	533.0	2.58	9174	0.780	17.87	22.10
F 1s	689.9	2.90	3266	1.000	0.24	0.35
Si 2p	102.2	2.53	1771	0.328	0.44	0.95

Table S14. C 1s spectrum of the PDA-GO_90 sample.

Peak	Position BE (eV)	FWHM (eV)	Raw Area (CPS)	Height	Atomic Conc %	Mass Conc %
C 1s *Ph*	284.63	1.06	3203	2754	24.93	24.93
C 1s *A*	285.00	1.24	2909	2137	22.63	22.63
C 1s *B*	285.71	1.23	2135	1583	16.61	16.61
C 1s *C*	286.47	1.23	2135	1583	16.61	16.61
C 1s *C1*	286.47	1.23	434	323	3.38	3.38
C 1s *Sh1*	287.70	1.80	1269	644	9.86	9.86
C 1s *Sh2*	289.16	1.80	528	268	4.10	4.10
C 1s *Sh3*	291.31	1.80	241	123	1.87	1.87

Table S15. N 1s spectrum of the PDA-GO_90 sample.

Peak	Position BE (eV)	FWHM (eV)	Raw Area (CPS)	Height	Atomic Conc %	Mass Conc %
N 1s *K*	398.77	1.54	220	131	11.66	11.66
N 1s *L*	400.19	1.54	1453	862	76.87	76.87
N 1s *M*	401.72	1.54	217	129	11.48	11.48

Table S16. Wide-scan spectrum of the PDA-GO_180 sample.

Peak	Position BE (eV)	FWHM (eV)	Raw Area (CPS)	RSF	Atomic Conc %	Mass Conc %
C 1s	284.9	2.23	10051	0.278	72.40	65.76
N 1s	399.9	1.51	1492	0.477	6.10	6.46
O 1s	532.7	2.37	7891	0.780	18.89	22.85
F 1s	689.1	2.51	10076	1.000	0.89	1.28
Si 2p	102.5	2.63	5659	0.328	1.72	3.65

Table S17. C 1s spectrum of the PDA-GO_180 sample.

Peak	Position BE (eV)	FWHM (eV)	Raw Area (CPS)	Height	Atomic Conc %	Mass Conc %
C 1s *Ph*	284.35	1.23	2956	2422	29.49	29.49
C 1s *A*	285.00	1.08	2515	2125	25.08	25.08
C 1s *B*	285.63	1.22	1687	1259	16.82	16.82
C 1s *C*	286.37	1.22	1687	1259	16.82	16.82
C 1s *C1*	286.37	1.22	0	0	0.00	0.00
C 1s *Sh1*	287.99	2.31	1059	418	10.55	10.55
C 1s *Sh2*	290.64	2.31	125	49	1.24	1.24

Table S18. N 1s spectrum of the PDA-GO_180 sample.

Peak	Position BE (eV)	FWHM (eV)	Raw Area (CPS)	Height	Atomic Conc %	Mass Conc %
N 1s *K*	398.51	1.53	174	103	11.59	11.59
N 1s *L*	400.05	1.53	1188	707	79.22	79.22
N 1s *M*	401.50	1.53	138	82	9.19	9.19
